# Integrating Senior Clinical Fellows Into a Nurse-Led Crisis Team: A Service Evaluation of In-Hours Medical Support

**DOI:** 10.1192/bjo.2026.11557

**Published:** 2026-06-30

**Authors:** Eileen Dempsey, Vivek Majumder

**Affiliations:** Royal Edinburgh Hospital, Edinburgh, United Kingdom

## Abstract

**Aims:**

To evaluate clinical activity, patient outcomes, and staff perceptions following integration of senior clinical fellows into a nurse-led crisis team.

**Methods:**

In August 2025, senior clinical fellows were embedded within the Royal Edinburgh Hospital’s Mental Health Assessment Service to provide in-hours medical support, previously covered ad-hoc by medics from other services.

A prospective activity log and a database of patients booked for medical follow-up after out-of-hours nursing assessment were maintained between 28^th^ September 2025 – 21^st^ January 2026 within the Mental Health Assessment Service. Staff feedback was obtained via an anonymous nursing survey.

**Results:**

Senior clinical fellows recorded 82 activities. There were 45 direct clinical contacts, mainly joint assessments with nursing staff, and indirect clinical contacts such as case discussions and GP liaison. Direct clinical contacts included both scheduled medical follow-up appointments and unscheduled reviews. The primary reasons for medical input were diagnostic clarity, risk assessment, prescribing, or Mental Health Act (MHA) decisions.

28 patients received 33 in-hours scheduled follow-ups, most commonly for suicidal ideation (n=14) or psychosis (n=10). Medication was initiated in 14 appointments (50%). Outcomes included signposting (n=10); referral to third sector services (n=5); referral to CommunityMental Health Teams (n=4) or Intensive Home Treatment Team (n=5); informal admission (n=2); admission under the MHA (n=2).

There were also unscheduled medical reviews according to clinical need. This included one patient detained by police and one further admission under the MHA.

Senior clinical fellows delivered 17 teaching and supervision sessions to nurses, foundation doctors, and students. They also formally supervised three nurses through a Clinical Assessment and Decision Making module for Advanced Nurse Practitioner training.

Seven nurses completed the survey. Prior to senior clinical fellows, reported time to access medical support for MHA assessments ranged from 30 to over 240 minutes. Post-implementation, access time ranged from immediate to under 60 minutes for all respondents. Reported benefits of senior clinical fellows included improved patient flow; reduced waiting times; enhanced confidence managing complex cases; and educational benefit. One postulated downside of senior clinical fellows was potential to disincentivise nurses to make complex decisions.

**Conclusion:**

Embedding senior clinical fellows in a nurse-led crisis team generated a range of clinical activity. It was associated with service enhancements to benefit patients, including diagnostic assessments and prescribing. Access to senior medical input was streamlined, with reduced delays and more efficient use of nursing time. There were also benefits for staff development. This model may be replicable across crisis services.

